# BRD4 contributes to the cytokine-induced acquisition of an IL-13RA2-positive inflammatory fibroblast phenotype

**DOI:** 10.1093/ecco-jcc/jjag128

**Published:** 2026-08-24

**Authors:** Rachele Frascatani, Mattia Alberto Serra, Andrea Iannucci, Marco Colella, Claudia Maresca, Irene Marafini, Elisabetta Lolli, Giorgia Sena, Andrea Divizia, Andrea Martina Guida, Giuseppe Sigismondo Sica, Giovanni Monteleone

**Affiliations:** Department of Systems Medicine, University of ‘Tor Vergata’, Rome, Italy; Department of Systems Medicine, University of ‘Tor Vergata’, Rome, Italy; Department of Biomedicine and Prevention, University of ‘Tor Vergata’, Rome, Italy; Department of Systems Medicine, University of ‘Tor Vergata’, Rome, Italy; Department of Systems Medicine, University of ‘Tor Vergata’, Rome, Italy; Department of Systems Medicine, University of ‘Tor Vergata’, Rome, Italy; Unità Operativa Complessa (UOC) Gastroenterologia, Azienda Ospedaliera Universitaria, Policlinico Tor Vergata, Rome, Italy; Unità Operativa Complessa (UOC) Gastroenterologia, Azienda Ospedaliera Universitaria, Policlinico Tor Vergata, Rome, Italy; Unità Operativa Complessa (UOC) Gastroenterologia, Azienda Ospedaliera Universitaria, Policlinico Tor Vergata, Rome, Italy; Department of Surgery, University of ‘Tor Vergata’, Rome, Italy; Department of Surgery, University of ‘Tor Vergata’, Rome, Italy; Department of Surgery, University of ‘Tor Vergata’, Rome, Italy; Department of Systems Medicine, University of ‘Tor Vergata’, Rome, Italy; Unità Operativa Complessa (UOC) Gastroenterologia, Azienda Ospedaliera Universitaria, Policlinico Tor Vergata, Rome, Italy

**Keywords:** Crohn’s disease, ulcerative colitis, mucosal inflammation, cytokines

## Abstract

**Background and Aims:**

Inflammatory bowel diseases (IBDs) are characterized by chronic intestinal inflammation, in which inflammatory fibroblasts contribute to epithelial damage and amplification of mucosal inflammation. However, the molecular mechanisms regulating their pathogenic activity remain incompletely understood. This study investigated the role of the epigenetic reader BRD4 in the differentiation and function of IL-13RA2-expressing inflammatory fibroblasts in IBD.

**Methods:**

Single-cell RNA sequencing datasets from IBD patients and controls were analyzed to define BRD4 expression across stromal populations. Protein expression was validated by immunofluorescence and flow cytometry in intestinal tissues and primary fibroblasts. Primary fibroblasts from IBD patients were stimulated with interleukin (IL)-4, IL-13, and tumor necrosis factor-alpha (TNF-α) to induce an inflammatory phenotype, and BRD4 function was assessed using the BRD4-targeting PROTAC AT1. Cytokine and chemokine expression was evaluated by RNA sequencing, real-time polymerase chain reaction (PCR), and ELISA, while neutrophil chemotaxis assays assessed the functional effects of fibroblast-derived mediators.

**Results:**

BRD4 was significantly upregulated in inflammatory fibroblasts from IBD patients compared with other stromal subsets and controls. Inflamed tissues showed enrichment of BRD4⁺ and IL-13RA2⁺ fibroblasts. In vitro, cytokine stimulation induced IL-13RA2 expression in the majority of fibroblasts, all of which co-expressed BRD4. BRD4 degradation significantly reduced the proportion of IL-13RA2⁺ fibroblasts. BRD4⁺ fibroblasts exhibited a pro-inflammatory transcriptional profile, including CCL2, CXCL1, CXCL2, IL-11, IL-24, IL-32, and CSF3. Degradation of BRD4 markedly decreased the expression of these mediators and reduced neutrophil migration.

**Conclusions:**

BRD4 contributes to cytokine-induced inflammatory activation and pro-inflammatory functions of intestinal fibroblasts in IBD. Targeting BRD4 may represent a potential strategy to modulate stromal cell-driven inflammation.

## 1. Introduction

Inflammatory bowel disease (IBD), which includes Crohn’s disease (CD) and ulcerative colitis (UC), is a chronic disorder of the gastrointestinal tract. The clinical course of IBD is usually unpredictable and marked by alternating periods of remission and relapse.^1^ During active phases, patients may experience symptoms (eg, abdominal pain, diarrhea, rectal bleeding, fatigue, and weight loss), which can significantly impair quality of life.^2^ Over time, ongoing inflammation may facilitate the development of local complications (eg, abscesses, toxic megacolon, colon cancer) and/or be associated with extraintestinal manifestations that can affect various organ systems, including the joints, skin, eyes, and hepatobiliary tract.^3–6^

IBD pathogenesis arises from a complex interplay among genetic predisposition, environmental exposures, alterations in the gut microbiota, and dysregulated immune responses.^7–9^ A central feature of both CD and UC is the breakdown of mucosal immune tolerance toward commensal microbes, leading to persistent and self-sustaining inflammation.^10–12^ Aberrant activation of innate immune cells, including macrophages, dendritic cells, and innate lymphoid cells, results in excessive production of pro-inflammatory cytokines such as tumor necrosis factor (TNF), interleukin (IL)-1β, IL-6, IL-12, and IL-23. These mediators drive the expansion of pathogenic T helper subsets, predominantly Th1 and Th17 cells in CD and atypical Th2-like responses in UC.^13^^,^^14^ At the same time, defective regulatory T cell function, impaired epithelial barrier integrity, and continuous microbial antigen exposure amplify adaptive T- and B- cell-driven immune responses.^15^ The resulting inflammatory environment promotes epithelial injury, leukocyte recruitment, and impaired tissue repair, creating a self-perpetuating cycle of mucosal damage.

Although immune cell dysregulation has long been considered the primary driver of IBD pathology, growing evidence highlights a critical role for stromal cells in sustaining intestinal inflammation. Intestinal fibroblasts are now recognized as active participants rather than passive structural elements. They sense inflammatory stimuli and directly shape immune responses within the tissue microenvironment.^16–18^ Single-cell transcriptomic studies have uncovered marked heterogeneity among intestinal fibroblasts, identifying distinct subsets associated with inflammatory signaling, extracellular matrix remodeling, and fibrotic progression.^19^^,^^20^ Persistent activation of myofibroblasts promotes excessive matrix deposition and contributes to fibrostenotic complications, particularly in CD. Interactions between TGF-β1-producing immune cells and stromal populations reinforce pro-fibrotic programs, linking chronic inflammation to irreversible tissue remodeling.^21^^,^^22^ In addition, specific fibroblast subsets adopt a pro-inflammatory phenotype characterized by the production of cytokines, chemokines, and matrix-degrading enzymes. These cells are primarily located in the lamina propria and subepithelial regions, where they closely interact with epithelial, endothelial, and infiltrating immune cells. Through the secretion of mediators such as IL-6, IL-8, IL-11, CCL2, and CXCL chemokines, inflammatory fibroblasts enhance leukocyte recruitment and sustain immune activation.^22–26^ They also respond to TNF and IL-17 signaling by upregulating adhesion molecules and survival factors, thereby facilitating immune cell retention within inflamed tissue.^27^^,^^28^ Collectively, these immune-stromal interactions play a central role in maintaining inflammatory activity and influencing long-term disease progression. A distinct fibroblast subset characterized by IL-13RA2 expression emerges in response to combined stimulation by TNF, IL-4, and IL-13. This population has been implicated in epithelial injury through prostaglandin E2-dependent mechanisms, further exacerbating barrier dysfunction.^29^ However, the molecular pathways that govern the differentiation and pathogenic activity of these IL-13RA2–expressing fibroblasts remain incompletely understood.

The epigenetic reader BRD4, a member of the BET protein family, might be a potential upstream regulator of this process. BRD4 coordinates cytokine-induced signaling with chromatin remodeling to control the transcription of genes involved in inflammation, cellular activation, and tissue remodeling. Notably, BRD4 expression is increased in IBD tissues across both immune and non-immune cell compartments and is associated with multiple pathogenic pathways.^30–33^ This study aimed to determine whether BRD4 regulates the differentiation and effector functions of IL-13RA2-expressing inflammatory fibroblasts.

## 2. Materials and methods

### Data source

Single-cell RNA sequencing (scRNA-seq) analyses were conducted using 2 publicly available datasets. Data for UC were retrieved from the Single Cell Portal of the Broad Institute of MIT and Harvard (https://singlecell.broadinstitute.org; accession SCP259) generated as part of the study by Smillie et al.^24^ Data for CD were obtained from the Single Cell Portal of the Broad Institute (accession SCP2959) generated by Kong et al.^19^

### Bioinformatic analyses

The following fibroblast subtypes were considered: WNT2B + Fos-lo 1, WNT2B + Fos-lo 2, WNT2B + Fos-hi, WNT5B + 1, WNT5B + 2, myofibroblasts, RSPO3+, and inflammatory fibroblasts for the UC dataset, and inflammatory fibroblasts, myofibroblasts, collagen-hi tissue fibroblasts, tissue fibroblasts, and SIP syncytium fibroblasts for the CD dataset.

Raw count matrices were normalized for library size using counts per ten thousand (CP10K), followed by log transformation [log(*x* + 1)]. For the CD dataset, normalization was performed using Scanpy (sc.pp.normalize_total, target_sum = 10⁴; sc.pp.log1p), ensuring consistency with the UC preprocessing workflow. Cells with zero total counts were excluded prior to normalization.

Dimensionality reduction was performed using a shared pipeline across datasets. The 10 000 most variable genes were selected from the normalized expression matrix based on variance. Truncated Singular Value Decomposition (TruncatedSVD) with 30 components was applied and used as input for Uniform Manifold Approximation and Projection (UMAP). In the CD dataset, the equivalent Scanpy workflow (highly variable gene selection, PCA, nearest-neighbor graph construction, and UMAP embedding) was applied.

#### Mucosal samples

Surgical specimens were collected from the inflamed colonic mucosa of 5 patients with UC (2 female; median age 57 years; range: 34-76) and 7 patients with CD (5 females; median age 57 years; range: 23-81). At the time of tissue collection, all patients had active disease. Three CD patients and 3 UC patients were not receiving therapy, whereas the remaining patients were treated with 5-aminosalicylic acid or biologic agents. Colonic mucosal biopsy samples were collected from 4 UC patients (3 female; median age 43 years; range: 30-73) and 5 CD patients (2 females; median age 57 years; range 49-73). All individuals had active disease at the time of collection. All patients were receiving 5-aminosalicylic acid and/or corticosteroids. Each patient who took part in the study gave written informed consent, and the study protocol was approved by the local ethics committees (Tor Vergata University Hospital, Rome, N RS 136.22).

#### Isolation and culture of intestinal fibroblasts

Unless otherwise indicated, all reagents were obtained from Sigma-Aldrich (Milan, Italy). Intestinal fibroblasts were isolated as previously indicated.^34^ Briefly, colonic specimens of patients with UC and CD were cultured in polystyrene flasks containing high-glucose DMEM supplemented with UltraGlutamine, 10% fetal bovine serum (FBS), 1% penicillin (100 U/mL), streptomycin (100 μg/mL), amphotericin B (2.5 μg/mL), 1% non-essential amino acids (all from Lonza, Verviers, Belgium), and 10 mM HEPES buffer. Cell outgrowth was typically observed after 2-4 weeks; cells were then expanded and used between passages 1 and 6. Fibroblasts were maintained in 75 cm² culture flasks at 37 °C in a humidified incubator with 5% CO₂. To induce an inflammatory phenotype, patient-derived fibroblasts (5 × 10⁴ cells/well) were seeded in 12-well plates, allowed to adhere for 24 h, and subsequently cultured either in medium alone (unstimulated, unst) or in the presence of IL-4 (10 ng/mL, Miltenyi Biotech, Bologna, Italy), IL-13 (10 ng/mL, Bio-Techne, Minneapolis, MN), and TNF-α (50 ng/mL, Bio-Techne, Minneapolis, MN) for the indicated time points. To investigate the role of BRD4 in regulating the inflammatory phenotype, IBD-derived fibroblasts were plated as described above and subjected to BRD4 inhibition either by transfection with a specific antisense oligonucleotide against BRD4 (BRD4 AS) or a scrambled control (NC AS) (2 µg/mL, Exiqon, Woburn, MA) for 72 h using Opti-MEM and Lipofectamine 3000 (Life Technologies, Milan, Italy) or by treatment with the BRD4-targeting PROTAC AT1 (1 µM, Bio-Techne, Minneapolis, MN), with dimethyl sulfoxide (DMSO) used as the vehicle control. During the final 48 h of BRD4 inhibition, cells were stimulated with IL-4, IL-13, and TNF-α.

#### Immunofluorescence

IBD and healthy control cryosections were fixed using 4% paraformaldehyde for 10 minutes. Sections were then washed with PBS 1X and permeabilized with 0.1% Triton-X for 20 minutes. Blocking procedure (PBS 1X, BSA 1%), was performed for 1 hour at room temperature and the slides were incubated with a mouse primary antibody anti-human BRD4 (1:100, Cell Signaling Technology, Danvers, MA, USA), rabbit primary antibody anti-human IL-13RA2 (1:50, Abcam, Cambridge, UK) and chicken primary antibody anti-human Vimentin (1:25, Invitrogen, Waltham, MA), overnight at 4 °C. After washing with PBS 1X, slides were incubated with a secondary antibody goat anti-rabbit Alexa488 (1:2000, Thermo Fisher Scientific, Waltham, MA), goat anti-mouse Alexa 568 (1:2000, Thermo Fisher Scientific, Waltham, MA) and goat anti-chicken Alexa 647 (1:200, Thermo Fisher Scientific, Waltham, MA) for 1 hour at room temperature. Slides were then washed, mounted using Prolong gold antifade reagent with DAPI (Thermo Fisher Scientific, Waltham, MA) and analyzed by Leica CTR6000 digital inverted microscope connected to a charge-coupled device camera and analyzed using the Leica software LAS-AF (Leica) for fluorescent microscopy (Leica, Wetzlar, Germany). Images were analyzed using ImageJ. A color threshold was applied to identify the fluorescent area corresponding to the co-localization of IL-13RA2, BRD4, and Vimentin. The percentage of co-localized positive area was calculated relative to the total Vimentin-positive area and expressed as IL-13RA2⁺BRD4⁺Vimentin⁺ area/Vimentin⁺ area × 100, as well as IL-13RA2⁺Vimentin⁺ and BRD4⁺Vimentin⁺ single-positive areas.

#### Real-time PCR

Total RNA was extracted from untreated fibroblasts, fibroblasts stimulated with IL-4, IL-13, and TNF-α treated or not with AT1 as indicated above, using the PureLink mRNA (Mini Kit Thermo Fisher Scientific, Waltham, MA). A constant amount of RNA (0.5 μg/sample) was retrotranscribed into complementary DNA (cDNA) as previously shown.^35^ The cDNA was then amplified by real-time polymerase chain reaction (PCR) using iTaq Universal SYBR Green Supermix (Bio-Rad Laboratories, Milan, Italy). Primers were as follows: human IL-13RA2: Fwd 5′-GTGGAGTGATAAACAATGCTGGG-3′, Rev 5′-TGGGTAGGTGTTTGGCTTACGC -3′; human CXCL1: Fwd 5′-AGCTTGCCTCAATCCTGCATCC-3′, Rev 5′-TCCTTCAGGAACAGCCACCAGT-3′; human CXCL2: Fwd 5′-GGCAGAAAGCTTGTCTCAACCC-3′, Rev 5′-CTCCTTCAGGAACAGCCACCAA -3′; human CCL2: Fwd 5′-AGAATCACCAGCAGCAAGTGTCC-3′, Rev 5′-TCCTGAACCCACTTCTGCTTGG -3′; human CXCL5: Fwd 5′-CAGACCACGCAAGGAGTTCATC-3′, Rev 5′-TTCCTTCCCGTTCTTCAGGGAG-3′; human IL11: Fwd 5′-GGACCACAACCTGGATTCCCTG-3′, Rev 5′-AGTAGGTCCGCTCGCAGCCTT-3′; human IL32: Fwd 5′-TCAAAGAGGGCTACCTGGAGAC-3′, Rev 5′-TCTGTTGCCTCGGCACCGTAAT-3′; human IL24: Fwd 5′-CTTCTCTGGAGCCAGGTATCAG-3′, Rev 5′-GGCACTCGTGATGTTATCCTGAG-3′; human CSF3: Fwd 5′-TCCAGGAGAAGCTGGTGAGTGA-3′, Rev 5′-CGCTATGGAGTTGGCTCAAGCA-3′; human β2M Fwd 5′-GTGGCCTTAGCTGTGCTC-3′, Rev 5′-AGAAAGACCAGTCCTTGCTG-3′; human COX-2 Fwd 5′-TTCTTGCCCAGCACTTCACGC-3′, Rev 5′-CTGTCTAGCCAGAGTTTCACCG-3′; human ACTA2 Fwd 5′-CTATGCCTCTGGACGCACAACT-3′, Rev 5′-CAGATCCAGACGCATGATGGCA-3′; human FAP Fwd 5′-GGAAGTGCCTGTTCCAGCAATG-3′, Rev 5′-GGAAGTGCCTGTTCCAGCAATG-3′. RNA expression was calculated relative to the housekeeping β-2 microglobulin gene using the Δ ΔCt algorithm.

##### Enzyme-linked immunosorbent assay

CXCL1 and CXCL2 protein levels were measured in the supernatants of fibroblasts treated as previously described. Quantification was performed using commercially available ELISA kits (Bio-Techne, Minneapolis, MN, USA) according to the manufacturer’s instructions. Absorbance was read at 450 nm using a multimode detector DTX 880 (Beckman Coulter, Milan, Italy).

#### Flow cytometry analysis

IBD fibroblasts were stained with LIVE/DEAD cell viability assay (Thermo Fisher Scientific, Waltham, MA). Cells were then washed and stained for 30 minutes at room temperature with anti-human IL-13RA2-VioBright V423 (Miltenyi Biotech, Bologna, Italy) or anti-human CD90 FITC (Miltenyi Biotech, Bologna, Italy). After washing, cells were fixed and permeabilized with Transcription Factor Staining buffer set (eBioscience, San Diego, CA) and Permeabilization Buffer (eBioscience, San Diego, CA), respectively. Cells were then stained with anti-Vimentin-PE (Miltenyi Biotech, Bologna, Italy) and BRD4 APC (Abcam, Cambridge, U.K.) for 30 minutes at room temperature. Appropriate isotype-matched controls were included. For the Annexin/propidium iodide (PI) assay, fibroblasts after treatment were collected, washed in Annexin V buffer, stained with FITC-Annexin V (final dilution: 1:100; Immunotools, Friesoythe, Germany), and with 5 mg/mL of PI for 30 minutes at room temperature. Gallios flow cytometer (Beckman Coulter, Brea, CA) was used for acquisition, and Kaluza software (Beckman Coulter) was used for analysis.

#### Neutrophil migration assay

Human neutrophils were isolated from peripheral whole blood using the MACSxpress Whole Blood Neutrophil Isolation Kit, human (Miltenyi Biotech, Bologna, Italy), according to the manufacturer’s instructions. Isolated neutrophils were resuspended in RPMI 1640 medium and used immediately for migration assays. Neutrophil migration was assessed using 24-well Transwell plates equipped with 8-µm pore size inserts (STEMCELL Technologies, Vancouver, Canada). Briefly, conditioned media were collected from inflammatory fibroblasts either stimulated with IL-4, IL-13, and TNF-α for 48 h or pretreated with AT1 for 24 h followed by stimulation with IL-4, IL-13, and TNF-α for further 48 h. Fibroblast-conditioned media were added to the lower chamber of each well.

A total of 5 × 10⁵ neutrophils suspended in 200 µL RPMI 1640 medium were seeded into the upper chamber of each Transwell insert. Cells were allowed to migrate for 2 h at 37 °C in a humidified incubator containing 5% CO₂. Following incubation, Transwell inserts were removed, and the medium from the lower chamber containing migrated neutrophils was collected. Migrated cells were recovered and counted using Trypan Blue exclusion to assess viable cells only. Results were expressed as the number of viable migrated neutrophils under each experimental condition.

### Statistical analysis

Data derived from the same donor under different experimental conditions were analyzed using a paired Student’s *t*-test. For analyses involving multiple group comparisons, a 2-way analysis of variance (2-way ANOVA) was performed. Statistical and graphical analyses were carried out using GraphPad Prism 9 (GraphPad Software, La Jolla, CA). *P*-values < 0.05 were considered statistically significant.

For dot plot analyses of bioinformatics data, mean log-normalized expression (including zero-expressing cells) and the percentage of expressing cells (expression > 0) were computed for each gene across stromal cell populations and conditions. These metrics were used for color and size encoding, respectively, and visualized using Seaborn based on pivot tables of cluster-condition combinations. Summary statistics included mean, median, standard deviation, and the percentage of expressing cells when appropriate.

BRD4 expression in inflammatory fibroblasts was visualized using violin plots with overlaid strip plots (jitter = 0.08-0.12, α = 0.35-0.40). Mean log-normalized BRD4 expression, including zero-expressing cells, was used to calculate the log2 fold-change between disease (CD or UC) and healthy inflammatory fibroblasts as the log2 ratio of the respective group means.

To further contextualize BRD4 expression, inflammatory fibroblasts were compared with immune cell populations within the same dataset using the same cluster-collapsing rules adopted for macro-group assignment. For each macro-group, the percentage of BRD4-expressing cells (expression > 0), together with the mean, median, and standard deviation of log-normalized expression were calculated. Pairwise comparisons between inflammatory fibroblasts and each macro-group were performed using 2-sided Mann–Whitney *U* tests, while overall differences across macro-groups within each compartment were assessed using the Kruskal–Wallis test.

For cytokine analyses, inflammatory fibroblasts were stratified into BRD4-high (upper quartile, Q4) and BRD4-low (lower quartile, Q1) groups based on BRD4 expression, while intermediate cells (Q2-Q3) were excluded from the analysis. A predefined panel of cytokine and chemokine genes was tested between groups using 2-sided Mann–Whitney *U* tests, followed by Benjamini–Hochberg false discovery rate (FDR) correction, with adjusted *P*-values < 0.05 considered statistically significant. For each gene, mean log-normalized expression, percentage of expressing cells, and log2 fold-change between BRD4-high and BRD4-low fibroblasts were calculated. Results were visualized as dot plots, with color representing mean expression and dot size representing the percentage of expressing cells; genes were ordered annotated according to statistical significance.

All analyses were conducted in Python 3. The main libraries used included Scanpy, SciPy, scikit-learn, umap-learn, NumPy, pandas, Matplotlib, and seaborn. These libraries were used for data processing, statistical analysis, dimensionality reduction, and data visualization, including UMAP embeddings, violin plots, dot plots, and expression heatmaps.

## 3. Results

### BRD4 is highly expressed in IBD inflammatory fibroblasts

**Figure 1.**
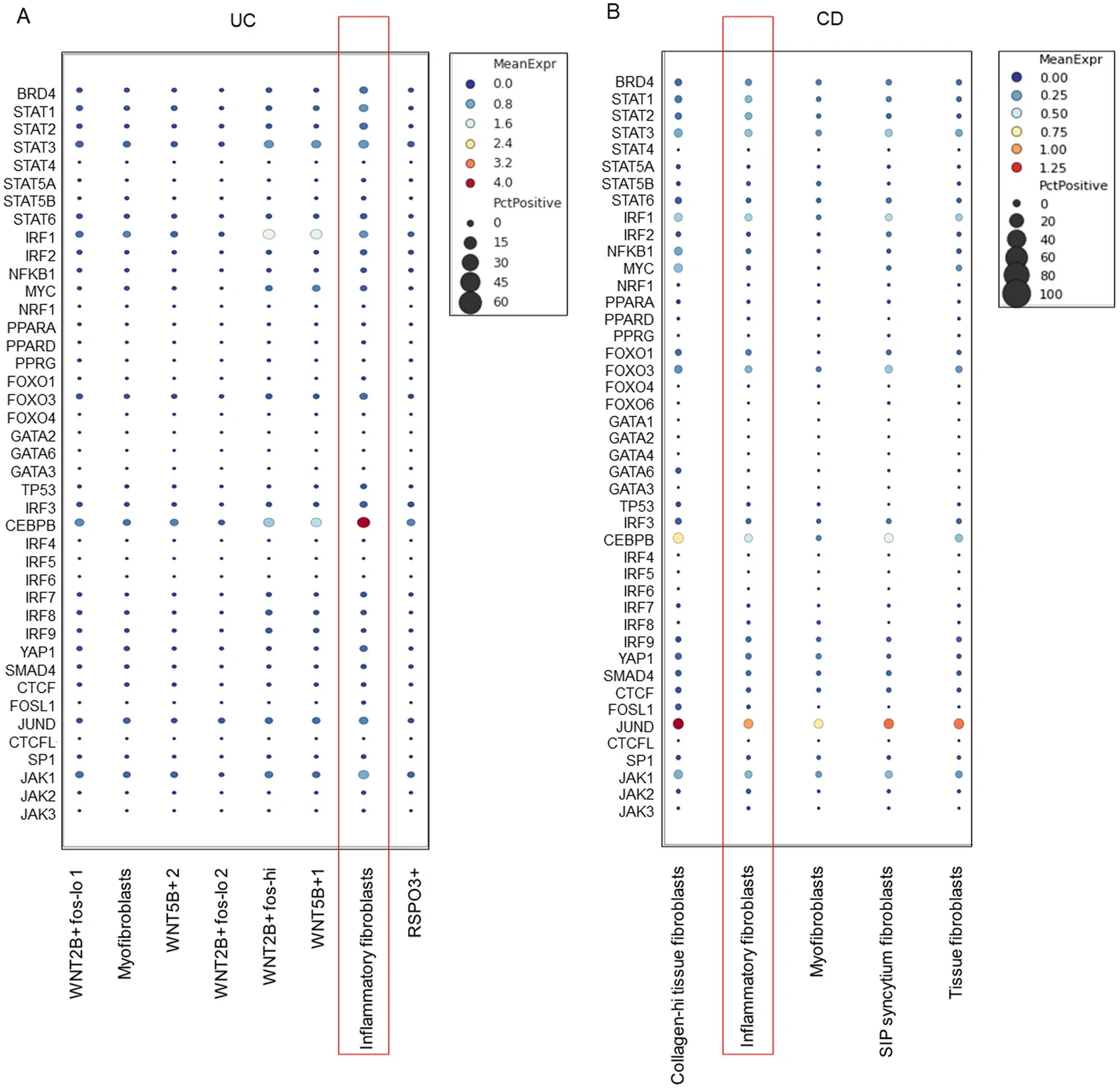
Transcription factor signatures across stromal cell populations in ulcerative colitis (UC) and Crohn’s disease (CD) single-cell RNA-seq datasets. (A) Dot plot showing transcription factor expression levels and the percentage of positive cells across distinct stromal cell clusters from UC patients (Single Cell Portal accession: SCP259). (B) Dot plot showing transcription factor expression levels and the percentage of positive cells across distinct stromal cell clusters from CD patients (Single Cell Portal accession: SCP2959).

**Figure 2.**
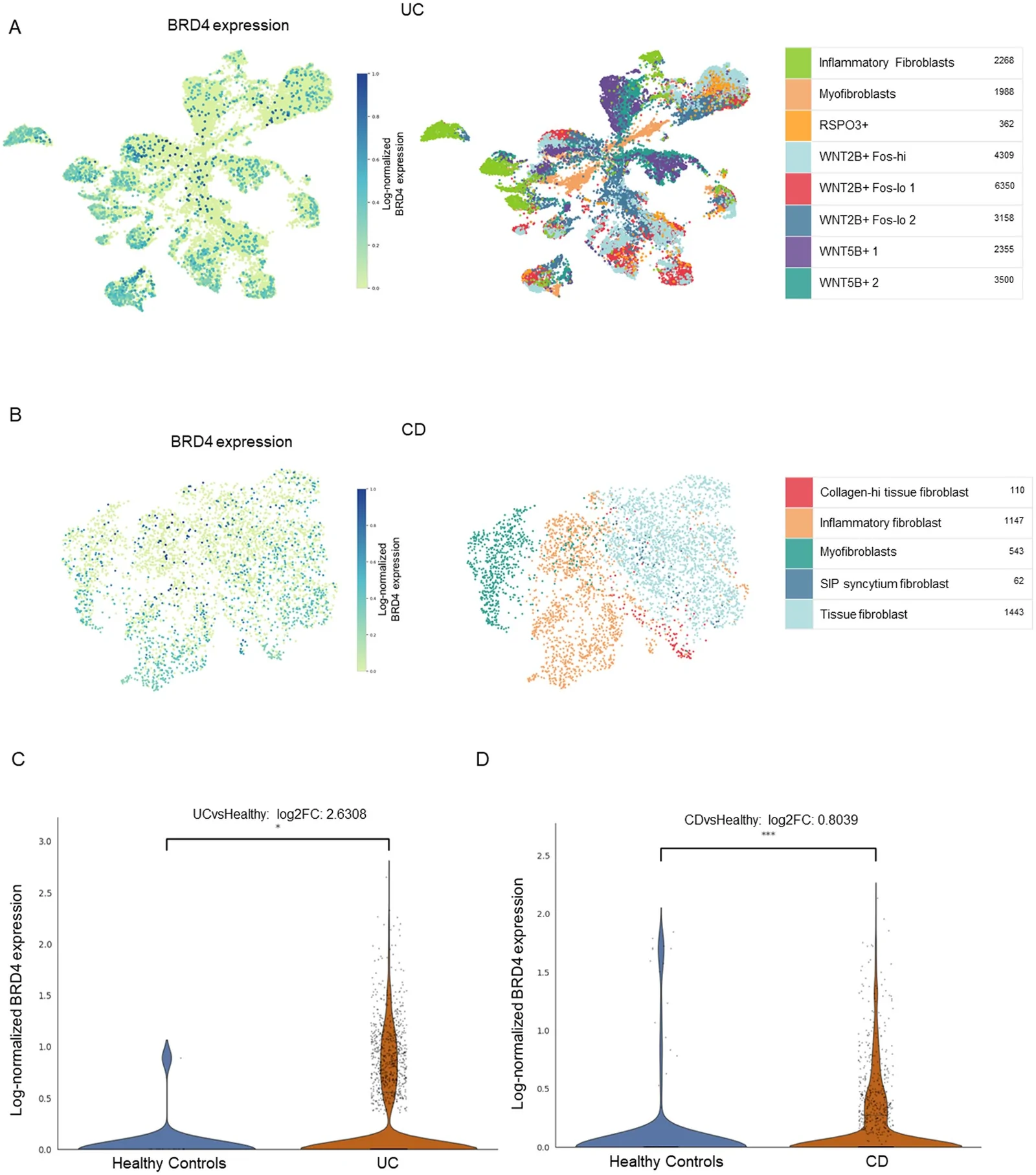
Up-regulation of BRD4 expression in inflammatory fibroblasts. (A) The Uniform Manifold Approximation and Projection (UMAP) plot illustrates the distribution of distinct stromal cell populations derived from ulcerative colitis (UC) patients (Single Cell Portal accession: SCP259), together with the corresponding UMAP feature plot showing BRD4 expression across the identified stromal subsets. (B) The UMAP plot illustrates the distribution of distinct stromal cell populations derived from Crohn’s disease (CD) patients (Single Cell Portal accession: SCP2959), together with the corresponding UMAP feature plot showing BRD4 expression across the identified stromal subsets. (C) The violin plot illustrates the log-normalized and the fold change of BRD4 expression. The analysis was performed on inflammatory fibroblasts from samples of UC and healthy controls (Single Cell Portal accession: SCP259). *P < 0.05. (D) The violin plot illustrates the log-normalized and the fold change of BRD4 expression. The analysis was performed on inflammatory fibroblasts from samples of CD and healthy controls (Single Cell Portal accession: SCP2959). ***P < 0.001.

We first examined whether inflammatory fibroblasts in IBD express BRD4. For this purpose, we analyzed publicly available scRNA-seq datasets from 2 independent IBD cohorts. The datasets were processed and analyzed using established scRNA-seq workflows to identify and characterize stromal cell populations based on canonical marker gene expression. Inflammatory fibroblasts from both UC and CD patients displayed similar transcriptional profiles, characterized by the upregulation of several transcription factors compared to other stromal cell populations (Figure 1). Notably, BRD4 RNA expression was significantly elevated in inflammatory fibroblasts from UC patients and CD patients relative to other stromal cell populations within the same patients (Figure 1A, B). UMAP analysis confirmed that, in UC, BRD4 RNA expression was more abundant in inflammatory fibroblasts than in other stromal cells (Figure 2A, Table 1). Consistently, the total number and percentage of BRD4-expressing cells, as well as the mean expression of BRD4 in single cells, were greater in inflammatory fibroblasts than in other stromal cell subsets (Table 1). Similar findings were observed in CD, with the exception that the percentage of BRD4-expressing inflammatory fibroblasts was higher than that of other stromal populations but lower than that of collagen-high tissue fibroblasts (Figure 2B, Table 2). Moreover, when the analysis was restricted to inflammatory fibroblasts, BRD4 expression was more pronounced in both CD and UC than in controls (Figure 2C, D). Furthermore, comparison of BRD4 expression across the major immune cell populations in the same public scRNA-seq datasets revealed that inflammatory fibroblasts expressed higher levels of BRD4 than many immune cell subsets, indicating that BRD4 upregulation is particularly enriched within the inflammatory stromal compartment rather than representing a general feature of inflamed tissues (Tables S1 and S2).

**Table 1.** BRD4 expression levels across stromal cell populations in ulcerative colitis derived from public single-cell RNA-seq datasets.

Cluster	Cells (*n*)	BRD4-positive cells (*n*)	BRD4-positive cells (%)	Mean expression
**Inflammatory fibroblasts**	2248	657	29.23	0.393
**WNT2B + Fos-hi**	2872	414	14.42	0.168
**WNT2B + Fos-lo 1**	4479	587	13.11	0.150
**RSPO3+**	203	22	10.84	0.133
**Myofibroblasts**	1649	178	10.79	0.139
**WNT5B + 1**	1406	150	10.67	0.124
**WNT5B + 2**	2330	210	9.01	0.102
**WNT2B + Fos-lo 2**	2398	154	6.42	0.071

**Table 2.** BRD4 expression levels across stromal cell populations in Crohn’s disease derived from public single-cell RNA-seq datasets.

Cluster	Cells (*n*)	BRD4-positive cells (*n*)	BRD4-positive cells (%)	Mean expression
**Collagen-hi tissue fibroblasts**	110	44	40.00	0.1042
**Inflammatory fibroblasts**	1163	396	34.05	0.1833
**Myofibroblasts**	545	129	23.67	0.1454
**SIP syncytium fibroblasts**	62	16	25.81	0.1517
**Tissue fibroblasts**	1445	338	23.39	0.1095

**Figure 3.**
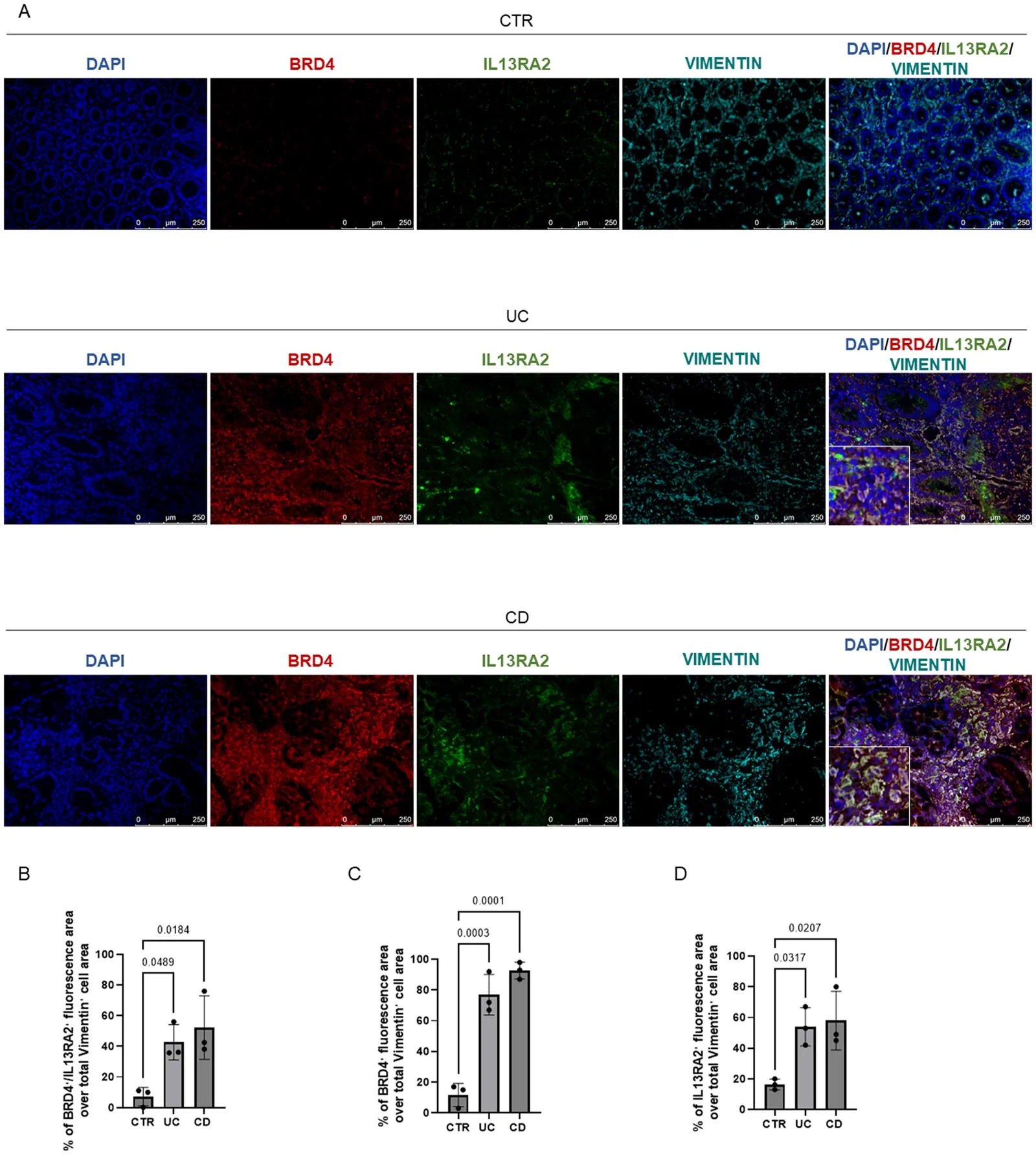
BRD4 and IL-13RA2 are co-expressed by intestinal fibroblasts in inflammatory bowel disease. (A) Representative immunofluorescence images for IL-13RA2 (green), BRD4 (red), vimentin (far red, shown in cyan), and DAPI (blue) of mucosal sections taken from a control subject (CTR) and inflamed tissues from one patient with ulcerative colitis (UC) and one patient with Crohn’s disease (CD). the inset in the merged images of UC and CD sections shows higher magnification. (B-D) Immunofluorescence images were analyzed using ImageJ. The percentages of IL-13RA2⁺BRD4⁺vimentin⁺ co-localized area, IL-13RA2⁺ area, and BRD4⁺ area were quantified by applying a color threshold and normalizing each fluorescent area to the total vimentin⁺ area (fluorescent area/vimentin⁺ area × 100).

To confirm such data at the protein level, we performed immunofluorescence on intestinal sections from IBD patients and normal controls, using antibodies targeting Vimentin, BRD4, or IL-13RA2. Fibroblasts positive for Vimentin and expressing BRD4 and/or IL-13RA2 were prevalent in IBD tissues, whereas these markers were scarcely present in the control samples (Figure 3).

Collectively, these data indicate that, in IBD, BRD4 is highly expressed by inflammatory fibroblasts.

### BRD4 expression during the differentiation of inflammatory fibroblasts

**Figure 4.**
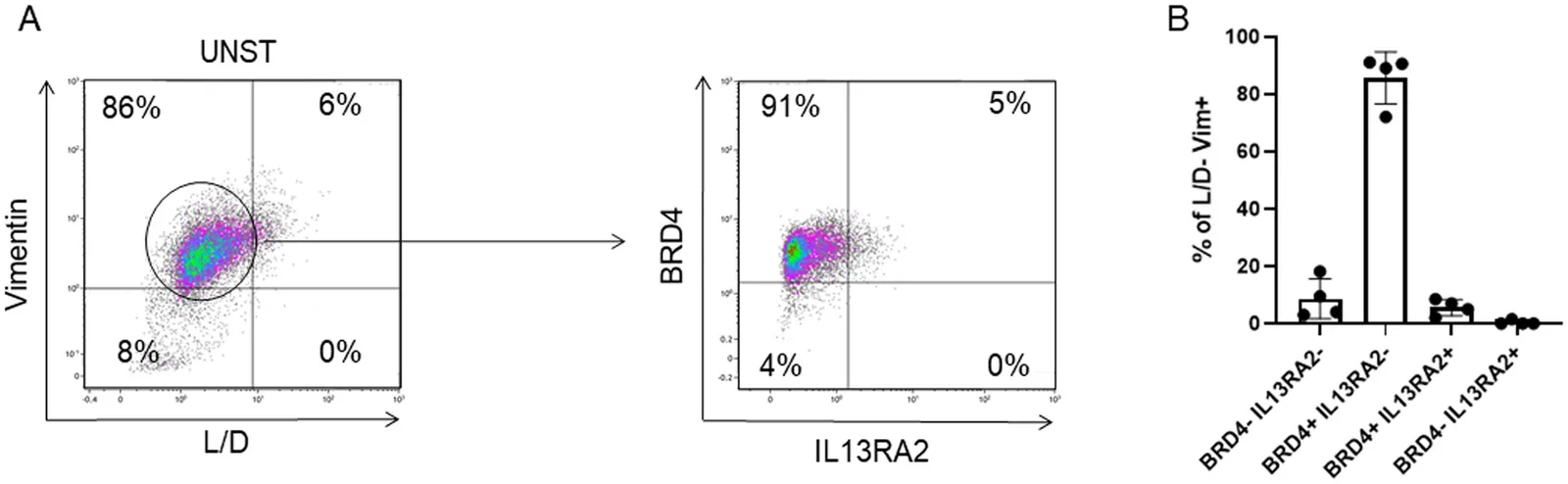
Intestinal fibroblasts isolated from IBD specimens and cultured in medium alone express BRD4 but lack IL-13RA2. (A) Representative dot plots showing the percentages of live Vimentin⁺ fibroblasts expressing BRD4 and/or IL-13RA2 as assessed by flow cytometry. (B). Histograms showing the percentages of positive cells. Data are presented as mean ± SD of 4 samples.

**Figure 5.**
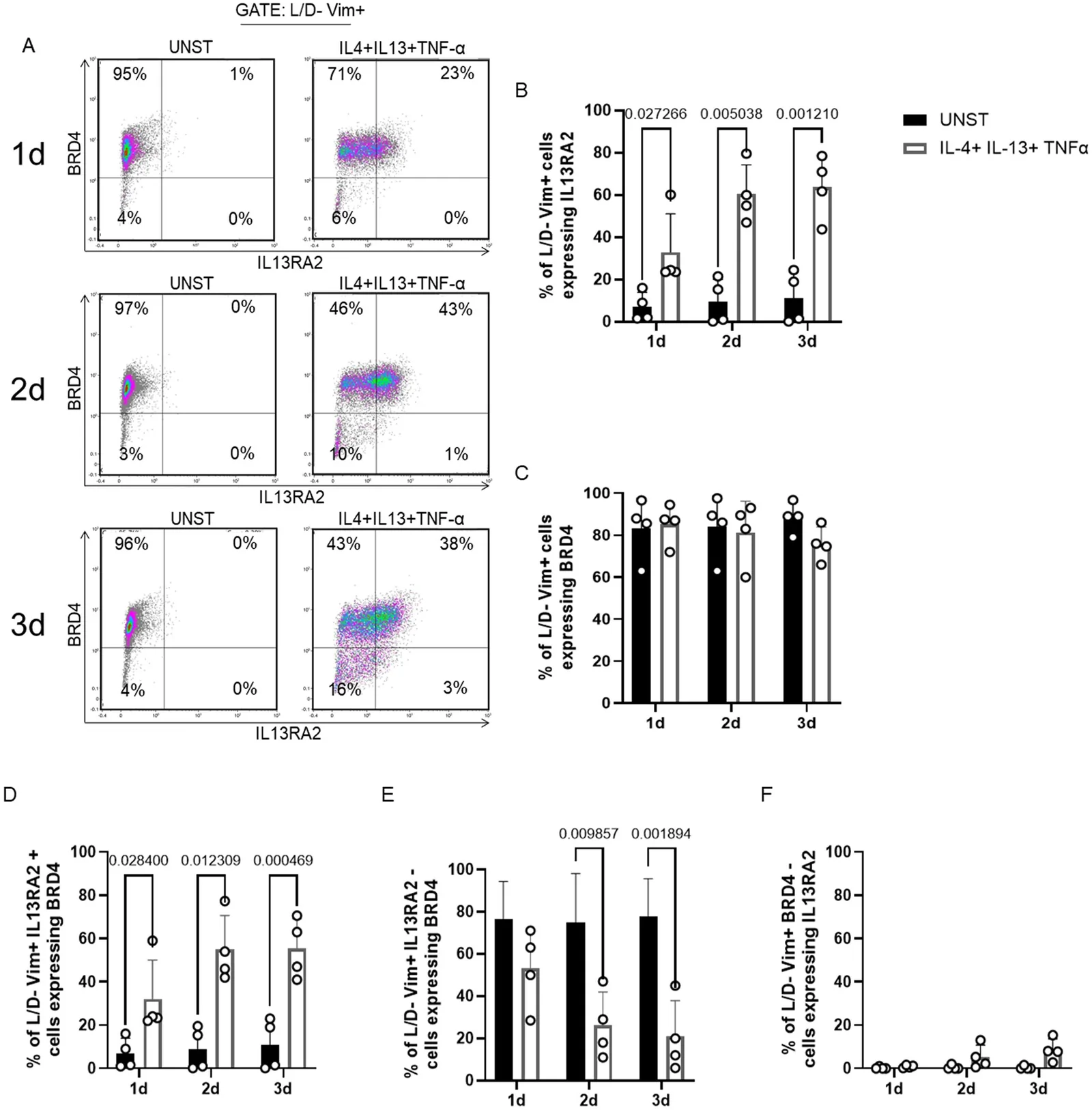
BRD4 Is highly expressed by cytokine-induced inflammatory fibroblasts. (A) Representative dot plots showing the percentages of (live +/dead −) Vimentin⁺ fibroblasts expressing BRD4 and/or IL-13RA2 as assessed by flow cytometry. Fibroblasts were isolated from intestinal mucosal samples of IBD patients and cultured with medium alone (unst) or with IL-4 (10 ng/mL), IL-13 (10 ng/mL), and TNF-α (50 ng/mL) for the indicated time points. (B-F). Histograms showing the percentages of live Vimentin⁺ fibroblasts expressing BRD4 and/or IL-13RA2 cultured as above. Data are presented as mean ± SD of 4 samples.

To evaluate the role of BRD4 in the differentiation of inflammatory fibroblasts, we isolated fibroblasts from IBD samples and cultured them for 1-6 passages. Cells expressed both Vimentin and CD90 (Figure S1), although downstream analyses were performed using Vimentin as the gating marker. We then assessed the expression of BRD4 and IL-13RA2 gating on live/dead, vimentin-positive cells by flow cytometry. More than 80% of the vimentin-positive cells expressed BRD4 (Figure 4A, B). Consistent with previous studies,^29^ less than 10% of fibroblasts cultured in medium alone expressed IL-13RA2 (Figure 4A, B). Notably, however, all the IL-13RA2-positive cells co-expressed BRD4. Time-dependent studies showed that the percentages of IL-13RA2-positive fibroblasts cultured in medium alone did not significantly change over time (Figure 4A, B). Stimulation of primary fibroblasts with IL-4, IL-13, and TNF-α induced a pronounced inflammatory phenotype, with the highest expression observed between days 2 and 3 of stimulation. At the later time point, more than two-thirds of the cells expressed IL-13RA2 (Figure 5A, B).

The proportion of BRD4-positive cells under unstimulated conditions remained stable over time (Figure 5A-C). Similarly, no significant change in the fraction of BRD4-positive cells was observed after cytokine stimulation compared to the unstimulated condition. Further analysis showed that cytokine stimulation significantly increased the percentage of cells co-expressing BRD4 and IL-13RA2, and this effect was seen at all time points (Figure 5A-D). In contrast, the proportion of IL-13RA2-negative BRD4-expressing cells decreased over time, with significant reductions evident at day 2 and 3 (Figure 5A, E). These findings suggest that cytokine stimulation drives a shift in BRD4-expressing cells from an IL-13RA2-negative state toward an inflammatory, IL-13RA2-positive phenotype. Additionally, cytokine stimulation slightly increased the fraction of BRD4-negative, IL-13RA2-expressing cells (Figure 5A, F). These latter data suggest that the induction of the inflammatory phenotype may involve factors beyond BRD4 in a small subset of fibroblasts.

### BRD4 is involved in the differentiation of inflammatory fibroblasts

**Figure 6.**
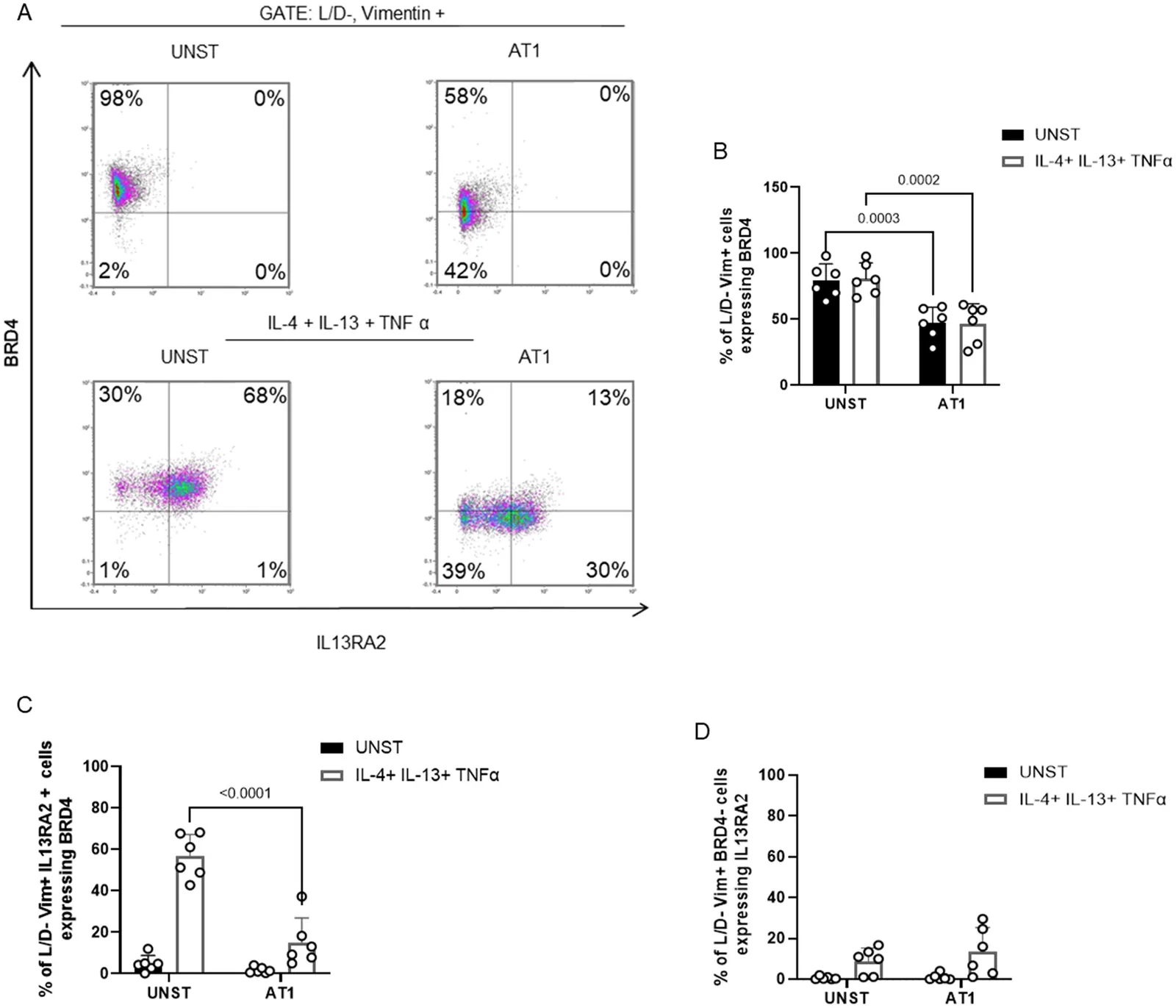
Pharmacological degradation of BRD4 reduces the percentage of vimentin⁺ fibroblasts expressing IL-13RA2. (A) Representative dot plots showing the percentages of (live +/dead −) Vimentin⁺ fibroblasts expressing BRD4 and/or IL-13RA2 as assessed by flow cytometry. Fibroblasts were isolated from intestinal mucosal samples of IBD patients and treated or not with a BRD4-specific PROTAC (AT1) for 72 hours. During the last 48 hours of culture, cells were either left unstimulated (Unst) or stimulated with IL-4, IL-13, and TNF-α. (B-D). Histograms show the percentages of live Vimentin⁺ fibroblasts expressing BRD4 and/or IL-13RA2 cultured as above. Data are presented as mean ± SD of 6 samples.

Given the strong association between IL-13RA2 expression and the presence of BRD4 under both unstimulated and cytokine-stimulated conditions, we explored the possibility that BRD4 acts as a positive regulator of the inflammatory phenotype. To test this, we degraded BRD4 using AT1, a heterobifunctional PROTAC molecule designed to selectively target BET family proteins, particularly BRD4, for proteasomal degradation.^36^ AT1 acts by recruiting BRD4 to the E3 ubiquitin ligase machinery, thereby promoting its ubiquitination and subsequent degradation. Prior to functional assays, AT1 was evaluated for cytotoxicity by Annexin V/PI staining, which showed no significant induction of apoptosis/necrosis in fibroblasts (Figure S3). For these experiments, cells were cultured in the presence or absence of AT1 for 72 hours. During the final 48 hours, cells were either left unstimulated or stimulated with IL-4, IL-13, and TNF-α. Following AT1 treatment, the percentage of BRD4-positive cells was significantly reduced compared to unstimulated conditions, regardless of cytokine stimulation (Figure 6A, B). Notably, functional degradation of BRD4 with AT1 significantly decreased the percentage of cells co-expressing IL-13RA2 and BRD4 (Figure 6C), while not affecting the fraction of BRD4-negative, IL-13RA2-expressing cells (Figure 6D). Similar results were observed when BRD4 expression was downregulated using a specific antisense oligonucleotide (Figure S4). These findings further support the role of BRD4 in the differentiation of the most inflammatory fibroblasts. To further confirm that the inflammatory phenotype was globally reversed and not solely linked to IL-13RA2 expression, we assessed additional fibroblast activation and inflammatory markers. In BRD4-degraded inflammatory fibroblasts compared with BRD4-expressing cells, real-time PCR analysis showed reduced expression of COX-2, FAP, and ACTA2 (Figure S5).

### BRD4 regulates the function of inflammatory fibroblasts

**Figure 7.**
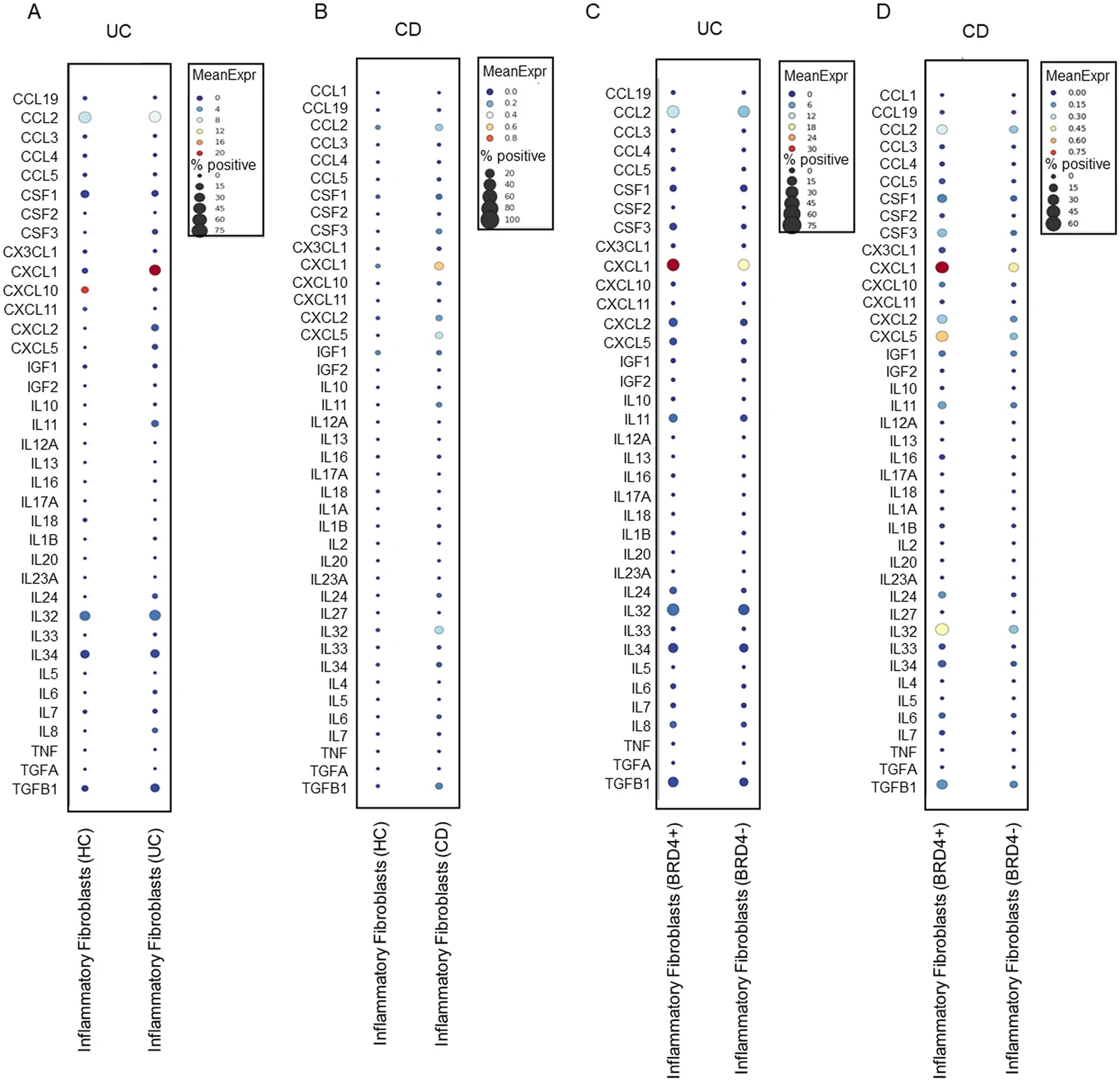
Expression of inflammatory cytokines and chemokines in BRD4-positive inflammatory fibroblasts in ulcerative colitis (UC) and Crohn’s disease (CD). (A) The dot plot illustrates cytokine and chemokine RNA expression levels and the percentage of cytokine/chemokine-positive inflammatory fibroblasts from healthy controls (HCs) and UC. (Single Cell Portal accession: SCP259). (B) The dot plot illustrates cytokine and chemokine RNA expression levels and the percentage of cytokine/chemokine-positive inflammatory fibroblasts from healthy controls (HC) and CD (Single Cell Portal accession: SCP2959). (C) The dot plot illustrates cytokine and chemokine RNA expression levels and the percentage of cytokine-positive inflammatory fibroblasts stratified by BRD4 expression status (BRD4⁺ and BRD4⁻) from UC (Single Cell Portal accession: SCP259). (D) The dot plot illustrates cytokine and chemokine RNA expression levels and the percentage of cytokine-positive inflammatory fibroblasts stratified by BRD4 expression status (BRD4⁺ and BRD4⁻) from CD samples (Single Cell Portal accession: SCP2959).

Next, we first examined the overall expression profile of cytokines and chemokines in inflammatory fibroblasts from CD and UC patients compared with healthy controls using the scRNA-seq datasets. This initial analysis revealed an increased inflammatory signature in IBD-associated inflammatory fibroblasts (Figure 7A, B). We then investigated which cytokines and chemokines are mainly associated with BRD4 expression within inflammatory fibroblasts.

**Figure 8.**
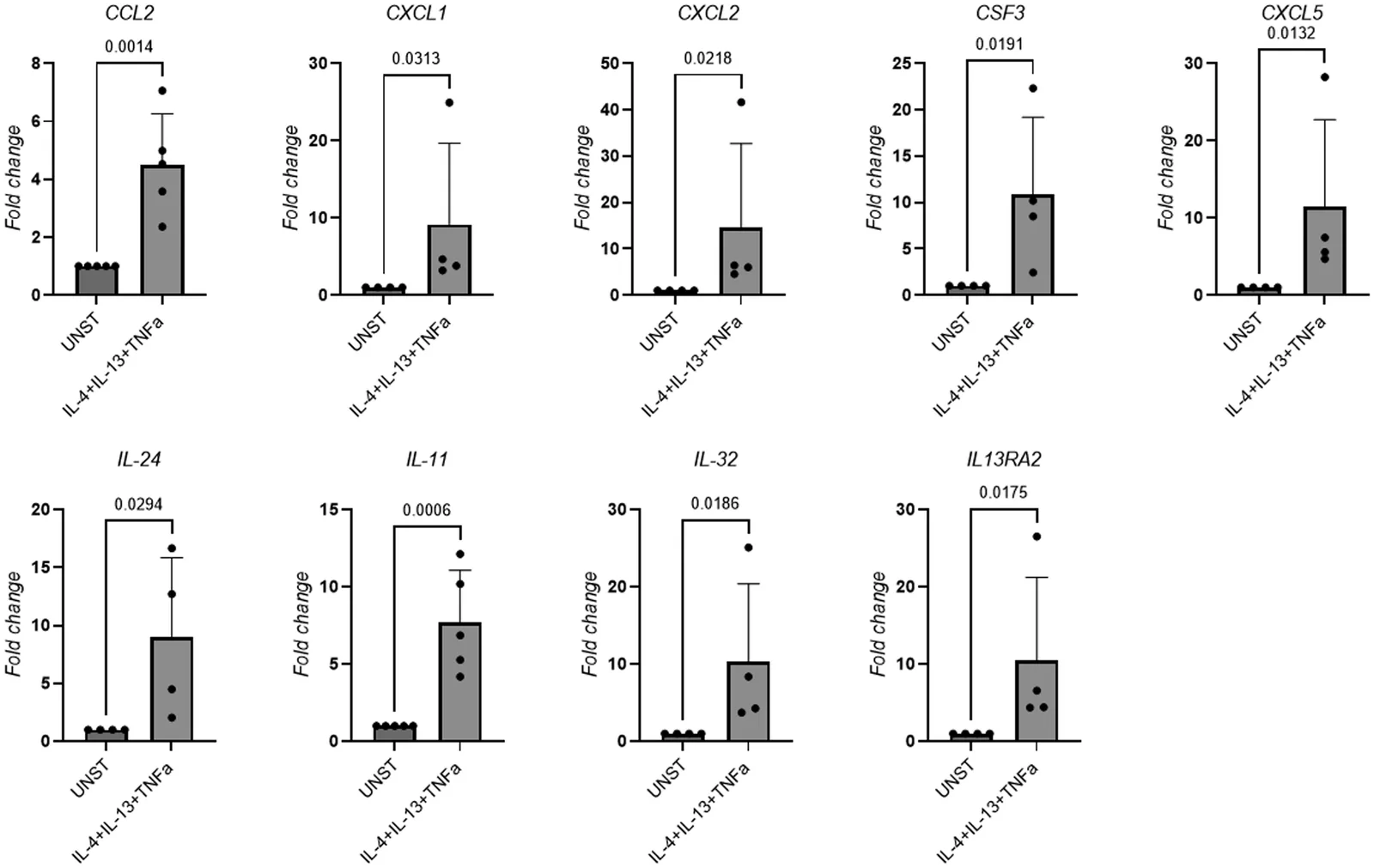
Inflammatory fibroblasts from IBD patients exhibit an upregulated cytokine and chemokine signature. (A) Fibroblasts isolated from intestinal mucosal samples of IBD patients were stimulated or not with IL-4, IL-13, and TNF-α for 72 hours. RNA transcripts of CCL2, CXCL1, CXCL2, CSF3, CXCL5, IL-24, IL-11, IL-32, and IL-13RA2 were evaluated by real-time polymerase chain reaction. Levels were normalized to β-2-microglobulin. Values represent mean ± SD of 5 independent experiments, each using fibroblasts from a different patient.

**Figure 9.**
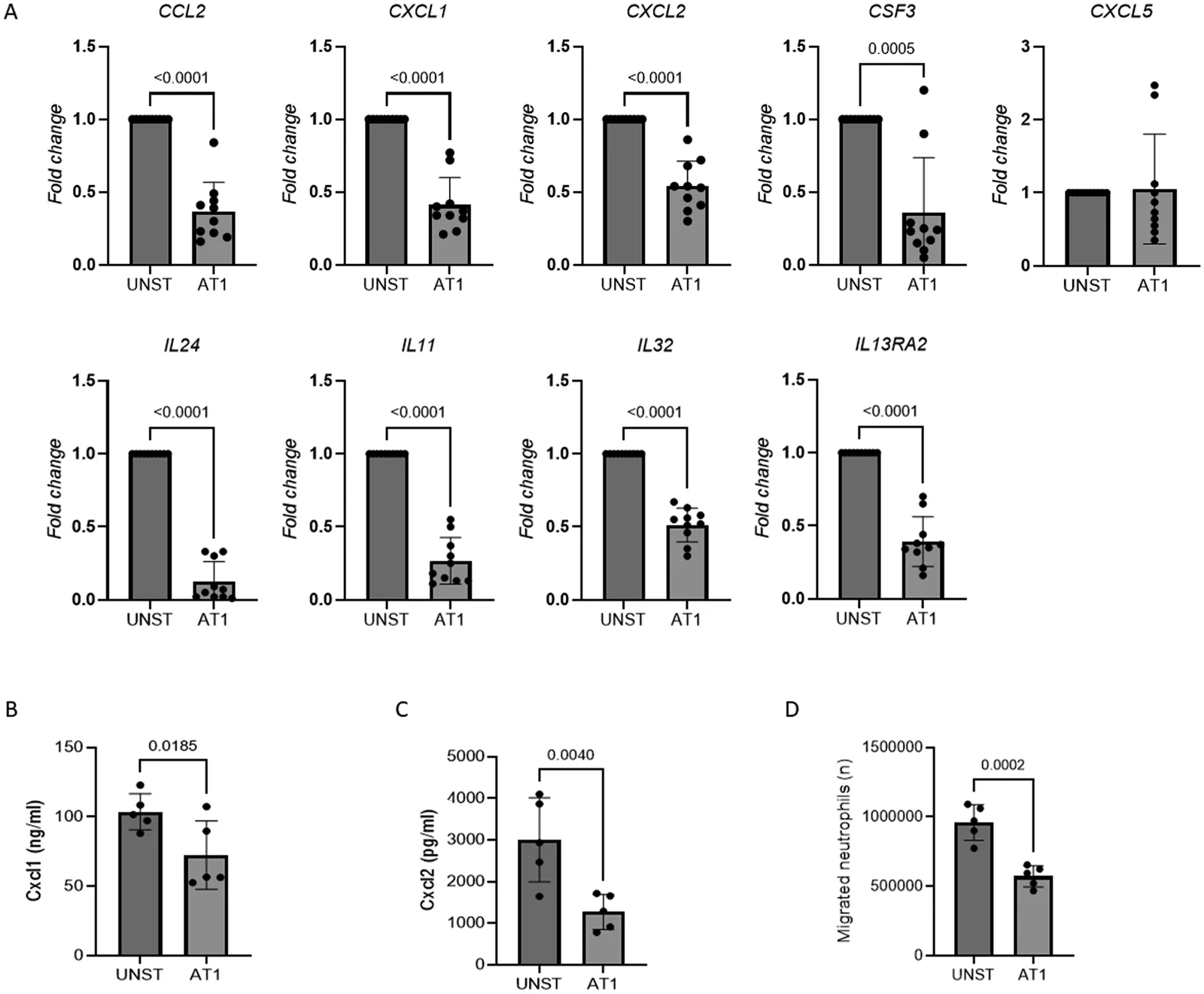
BRD4 Degradation dampens inflammatory cytokine and chemokine production and decreases fibroblast-mediated neutrophil chemotaxis in intestinal inflammatory fibroblasts from IBD patients. (A) Fibroblasts isolated from intestinal mucosal samples of IBD patients were treated or not with a BRD4-specific PROTAC (AT1) for 72 hours. During the last 48 hours of culture, cells were stimulated with IL-4, IL-13, and TNF-α. RNA transcripts of CCL2, CXCL1, CXCL2, CSF3, CXCL5, IL-24, IL-11, IL-32, and IL-13RA2 were evaluated by real-time polymerase chain reaction. Levels were normalized to β-2-microglobulin. Values represent mean ± SD of 10 independent experiments, each using fibroblasts from a different patient. (B, C) CXCL1 and CXCL2 protein levels in culture supernatants collected from intestinal inflammatory fibroblasts treated as described in panel A were quantified by ELISA. Values represent mean ± SD of 5 independent experiments. (D) Neutrophil chemotaxis assay performed using a Transwell system. Human neutrophils were seeded in the upper chamber, while supernatants collected from intestinal inflammatory fibroblasts treated as described in panel A were placed in the lower chamber as chemoattractant. After incubation, migrated neutrophils were quantified, and results are expressed as number of migrated cells. Data indicate mean ± SD from 5 independent experiments, each using fibroblasts from a different patient.

To this end, we compared BRD4-positive versus BRD4-negative inflammatory fibroblast populations in IBD, showing that BRD4-positive cells expressed a broad array of cytokines and chemokines, including CCL2, CXCL1, CXCL2, CXCL5, IL-11, IL-24, IL-32, and colony-stimulating factor 3 (CSF3) (Figures 7C, D and S6). Real-time PCR confirmed that cytokine-stimulated inflammatory fibroblasts exhibited high levels of these mediators (Figure 8). To evaluate whether BRD4 regulates the inflammatory function of these cells, cytokine-stimulated fibroblasts were treated with AT1, and the expression of these molecules was assessed by real-time PCR. BRD4 downregulation was associated with reduced expression of all these inflammatory factors, with the exception of CXCL5 (Figure 9A). CXCL1 and CXCL2 protein levels were further quantified by ELISA (Figure 9B, C), confirming their reduction upon BRD4 silencing in inflammatory fibroblasts. Accordingly, functional chemotaxis assays using neutrophils and fibroblast-conditioned medium demonstrated that BRD4 knockdown significantly reduced neutrophil migration (Figure 9D).

Overall, these data demonstrate that BRD4 is required to maintain the transcriptional program of pro-inflammatory mediators, reinforcing its central role in sustaining the inflammatory fibroblast state in IBD.

## 4. Discussion

Understanding the origins, regulation, and functional diversity of inflammatory fibroblasts, along with the complex immune networks involved in IBD, provides critical insights into disease mechanisms and potential therapeutic approaches. This study was undertaken to investigate the role of BRD4 in sustaining the inflammatory phenotype of fibroblasts, particularly IL-13RA2-expressing fibroblasts, within the context of IBD. Although these cells are believed to contribute to epithelial damage and barrier dysfunction through prostaglandin E2-dependent pathways,^29^ the precise molecular mechanisms driving their differentiation and pathogenic behavior are not yet fully understood.

Our findings show that BRD4 expression is significantly elevated in inflammatory fibroblasts from both CD and UC patients compared to healthy controls. The most striking finding was the particularly high BRD4 levels in UC inflammatory fibroblasts relative to other stromal cell populations within the same patients, highlighting its potential role in promoting fibroblast differentiation in response to inflammatory signals in the gut. Interestingly, although BRD4 expression was elevated in inflammatory fibroblasts compared to myofibroblasts and SIP syncytium fibroblasts in CD, it was lower in comparison to collagen-high tissue fibroblasts. This suggests that BRD4 may be more specifically involved in the activation of certain fibroblast subsets in the inflamed tissue, supporting previous studies indicating its key role in promoting fibrogenesis across various pathological conditions.^33^

To further investigate BRD4's functional role in inflammatory fibroblast differentiation, we isolated primary fibroblasts from IBD patients and cultured them under varying conditions. In line with previous studies, cultured fibroblasts gradually lost IL-13RA2 expression, whereas BRD4 expression remained stable over time.^29^ This suggests that, in the absence of inflammatory signals, BRD4 alone is insufficient to induce or maintain the inflammatory fibroblast phenotype. However, when cultured fibroblasts from IBD patients were exposed to pro-inflammatory cytokines (eg, IL-4, IL-13, and TNF-α), they re-expressed IL-13RA2. Notably, BRD4 degradation resulted in a marked reduction of this inflammatory phenotype, highlighting BRD4’s crucial role in fibroblast differentiation in response to cytokine signals.

While the precise molecular pathways driving IL-13RA2 expression in inflammatory fibroblasts remain elusive, it is likely that activation of key transcription factors induced by IL-4, IL-13, and TNF-α such as Stat proteins, AP-1, and NF-κB, plays a critical role. As BRD4 is known to interact with and regulate these transcription factors in various cell types,^37–40^ it is plausible that BRD4's regulation of inflammatory gene expression in fibroblasts may involve these pathways, at least in part, although this remains speculative in our system and requires direct experimental validation. To explore how BRD4 activity impacts fibroblast function, we analyzed cytokine and chemokine profiles of BRD4-positive and BRD4-negative fibroblasts from IBD patients using scRNA-seq data. BRD4-positive fibroblasts expressed a wide range of pro-inflammatory cytokines and chemokines essential for recruiting immune cells to sites of inflammation and amplifying tissue damage. One such molecule is CSF3, a member of the IL-6 superfamily whose receptor is primarily expressed on neutrophils and inhibits neutrophil apoptosis both in vivo and in vitro.^41^^,^^42^ Indeed, receptor–ligand analysis showed that inflammatory fibroblasts have the ability to recruit and maintain neutrophils and monocytes through the CSF3-CSF3R axis.^43^ Furthermore, a gene expression analysis associated with unresponsiveness to anti-TNF therapy in UC revealed over-expression of IL-13RA2, IL-11, IL-24, and CSF3, all of which are up-regulated by inflammatory fibroblasts.^44^ Consistently, IL-13RA2 expression has been identified as a predictive biomarker for the response to anti-TNF antibodies in both UC and CD.^6^^,^^45^ Degradation of BRD4 in cytokine-stimulated fibroblasts from IBD patients led to a significant decrease in the expression of these molecules, reinforcing the importance of BRD4 in regulating fibroblast function during inflammation. Importantly, the reduction of CXCL1 and CXCL2 following BRD4 degradation was further confirmed at the protein level by ELISA. Moreover, conditioned medium from BRD4-degraded inflammatory fibroblasts exhibited a significantly reduced capacity to promote neutrophil chemotaxis, demonstrating that the transcriptional changes induced by BRD4 inhibition translate into a functional impairment of neutrophil recruitment. These findings provide functional evidence that BRD4 regulates the production of neutrophil-attracting chemokines by inflammatory fibroblasts, thereby contributing to the amplification of intestinal inflammation.

These findings extend previous research on the role of BRD4 in the control of fibroblast activity within inflammatory contexts. For instance, pan-BET inhibitors have been shown to suppress the activation and proliferation of fibroblast-like synoviocytes in rheumatoid arthritis ^46^ and to modulate pro-inflammatory gene expression in cardiac fibroblasts by inhibiting BRD4’s positive regulation of NF-κB-mediated inflammation.^47^ Our results also align with studies demonstrating that BRD4 positively regulates the production of several chemokines in various inflammatory models.^48–50^ Nevertheless, these findings should be interpreted in light of some limitations of our study, including the relatively limited number of patient-derived samples analyzed and the lack of in vivo validation.

In conclusion, this study underscores the critical role of BRD4 in the differentiation and function of inflammatory fibroblasts in IBD. By elucidating BRD4’s involvement in maintaining the inflammatory phenotype and modulating cytokine production, our findings open new avenues for therapeutic strategies aimed at targeting fibroblast-driven inflammation in IBD. However, given the ubiquitous expression of BRD4 and its broad physiological functions, systemic BRD4 inhibition may produce effects beyond intestinal fibroblast function. Therefore, future studies should explore cell-selective or locally delivered therapeutic approaches to maximize efficacy while minimizing off-target effects. Further research is needed to fully understand the mechanisms by which BRD4 coordinates these processes and interacts with other signaling pathways.

## Supplementary Material

jjag128_Supplementary_Data

## Data Availability

Some data underlying this article are available in the article and in its online supplementary material. Other data underlying this article were obtained from the Single Cell Portal of the Broad Institute of MIT and Harvard (https://singlecell.broadinstitute.org; accession numbers SCP259 and SCP2959). The derived data generated in this research will be shared on reasonable request to the corresponding author. The code used for the scRNA-seq analyses is also available upon reasonable request from the corresponding author.
