# Supplementary material for: BRD4 contributes to the cytokine-induced acquisition of an IL-13RA2-positive inflammatory fibroblast phenotype
Source: J Crohns Colitis. 2026 Aug 24;20(8):jjag128. doi: 10.1093/ecco-jcc/jjag128 (PMC13501532; doi:10.1093/ecco-jcc/jjag128)
Supplement: Supplementary file 1 [file jjag128_supplementary_data.zip › Supp..odt]

Supplementary Figure 1: (A) Representative dot plots showing CD90 and Vimentin expression in fibroblasts analyzed in live/dead cells by flow cytometry. Fibroblasts were isolated from patient-derived samples and either treated or not with the BRD4 PROTAC AT1 for 72 hours. During the last 48 hours of culture, cells were either left unstimulated (Unst) or stimulated with IL-4, IL-13, and TNFα to induce an inflammatory phenotype. (B) Histograms showing the percentages of live CD90⁺ Vimentin⁺ fibroblasts across the indicated experimental conditions. (C) Representative dot plots illustrating the induction of the inflammatory fibroblast phenotype and the efficiency of BRD4 PROTAC AT1. Data are representative of three independent experiments.

Supplementary Figure 2: Representative FACS plots showing the gating strategy, including unstained and isotype controls, used for the analysis of inflammatory fibroblast samples.


Supplementary Figure 3: AT1 treatment does not affect the viability of inflammatory fibroblasts
(A) Representative Annexin V/propidium iodide dot plots of inflammatory fibroblasts treated with vehicle (DMSO) or the BRD4 PROTAC AT1 for 72 hours. IL-4, IL-13, and TNF-α were added to the cultures in the last 48 hours. Staurosporine-treated cells were used as a positive control for cell death. (B–E) Quantification of the percentages of Annexin V⁺/Propidium (PI)⁺ (late apoptotic/dead), Annexin V⁺/PI⁻ (early apoptotic), Annexin V⁻/PI⁺ (necrotic), and Annexin V⁻/PI⁻ (viable) cells under the indicated experimental conditions. Data are representative of three independent experiments.


Supplementary Figure 4: BRD4 knockdown reduces the percentage of Vimentin⁺ fibroblasts expressing IL-13RA2
(A) Representative dot plots showing the percentages of (live/dead -) Vimentin⁺ fibroblasts expressing BRD4 and/or IL-13RA2 as assessed by flow cytometry. Fibroblasts were isolated from intestinal mucosal samples of IBD patients and transfected with either a scrambled control oligonucleotide (s-AS) or a BRD4-specific antisense oligonucleotide (BRD4 AS) for 72 hours. During the last 48 hours of culture, cells were either left unstimulated (Unst) or stimulated with IL-4, IL-13, and TNFα. (B–D) Histograms showing the percentages of live Vimentin⁺ fibroblasts expressing BRD4 and/or IL-13RA2 cultured as above. Data are presented as the mean ± SD of five samples. 


Supplementary Figure 5: RNA expression of inflammatory cytokines and chemokines in BRD4-positive inflammatory fibroblasts in ulcerative colitis (UC) (C) and Crohn’s disease (CD) (D).  Cells were stratified into quartiles based on BRD4 expression, and the first and fourth quartiles were compared. Statistical significance is reported for differentially expressed cytokines and chemokines reaching statistical significance (Single Cell Portal accession numbers: SCP259 and SCP2959). 

Supplementary Figure 6: Fibroblasts isolated from intestinal mucosal samples of IBD patients were treated or not with a BRD4-specific PROTAC (AT1) for 72 hours. During the last 48 hours of culture, cells were stimulated with IL-4, IL-13, and TNFα. RNA transcripts of COX2, FAP, and ACTA2 were evaluated by real-time
polymerase chain reaction. Levels were normalized to β-2-microglobulin. Values represent
mean ± SD of five independent experiments, each using fibroblasts from a different
patient.

Supplementary Table 1: BRD4 expression across immune cell populations compared to inflammatory fibroblasts (UC)
MACRO_GROUP
CELLS (n)
BRD4-POSITIVE CELLS (%)
MEAN EXPRESSION
p-value
Inflammatory Fibroblasts
2248
29,23
0,2764
—
Dendritic cells
1946
24,41
0,242
p < 0.01
GC B cells
761
16,03
0,2195
p < 0.001
ILCs
356
10,67
0,1966
p < 0.001
NK cells
1577
10,59
0,1952
p < 0.001
Inflammatory Monocytes
1529
9,88
0,1633
p < 0.001
CD8+ IL17+
392
9,69
0,1468
p < 0.001
Macrophages
13107
9,67
0,1483
p < 0.001
Regulatory T cells
5425
9,07
0,1604
p < 0.001
Plasma cells
60573
8,24
0,0881
p < 0.001
Follicular B cells
17846
8,07
0,1408
p < 0.001
CD4+ Activated Fos-lo
5862
8,03
0,1338
p < 0.001
CD8+ LP
11295
7,84
0,1433
p < 0.001
CD4+ Activated Fos-hi
6882
7,44
0,1282
p < 0.001
CD8+ IELs
2054
7,16
0,1246
p < 0.001
CD4+ Memory
21816
7,14
0,1256
p < 0.001
CD4+ PD1+
342
6,14
0,0903
p < 0.001
Mast cells
4807
4,29
0,0813
p < 0.001


Supplementary Table 2: BRD4 expression across immune cell populations compared to inflammatory fibroblasts (CD)
MACRO_GROUP
CELLS (n)
BRD4-POSITIVE CELLS (%)
MEAN EXPRESSION
p-value
Inflammatory Fibroblasts
1163
34,05
0,1833
—
M2-like Macrophages
1928
49,95
0,2031
p < 0.001
CD8 T cells
1520
46,25
0,1659
p < 0.001
GZMB-hi B cells
355
45,35
0,2384
p < 0.001
Dendritic cells
1591
44,75
0,1657
p < 0.001
Lyve1 Macrophages
229
40,17
0,1973
ns
Monocytes
5059
33,27
0,1677
ns
CXCR6-hi CD4 T cells
2265
22,69
0,1443
p < 0.001
CD69+ CD4 T cells
649
22,65
0,1427
p < 0.001
Intraepithelial Tgd
1723
20,14
0,1274
p < 0.001
LP-associated NK/Tgd
2403
18,64
0,1215
p < 0.001
Follicular helper T cells
2498
17,77
0,1182
p < 0.001
B cells
22004
17,27
0,1187
p < 0.001
Plasma cells
28043
17,24
0,0404
p < 0.001
Regulatory T cells
5497
17,06
0,1274
p < 0.001
GZMK-hi CD8 T cells
4709
13,65
0,1054
p < 0.001
CCR7hi T cells
12525
13,3
0,0937
p < 0.001
PLGC2-hi T cells
933
12,43
0,1338
p < 0.001
CD4 T cells
9518
12,11
0,1029
p < 0.001
Tissue Macrophages
2109
11,95
0,0832
p < 0.001
CD8 Intraepithelial cells
4935
10,72
0,0886
p < 0.001
T cells
3233
7,83
0,0638
p < 0.001
